# Progress in Diseases of the Heart

**Published:** 1900-10-20

**Authors:** 


### Progress in Diseases of the Heart.

(*Continued from page* 35.)

**Heart Sounds.**—Creighton[10] gives his conclusions, after the study of 1,000 cases, that as regards the relative intensity of the second cardiac sounds at the base, (1) the accentuation of the pulmonic second sound is almost invariable in young children, and frequent in youth. (2) After the 40th year the reverse is the case, it being rare to find the pulmonic second sound as loud as the corresponding aortic sounds. (3) Between 20 and 30 there is no marked accentuation of either sound.

Jacobi[11] considers that functional heart murmurs without definite pathological changes in the heart are rare. The murmurs heard in children are due to cardiac insufficiency, inasmuch as the cardiac walls do not develop proportionately to the cardiac cavities, and remain thinner than normal.

Brockbank[12] records several cases of heart disease where murmurs, mitral or aortic in origin, were audible in unusual situations and at considerable distances. Having noticed a mitral murmur audible in the lumbar region (p.-m. no other cause for murmur), he examined systematically cases of cardiac disease, and has collected 24 patients in whom a murmur of mitral or aortic origin could be heard over spleen, liver, whole of chest anteriorly and posteriorly, carotid arteries and abdominal aorta, the vertebral column from 4th D. vert. to sacrum, and the right and left renal regions. No pressure was made with the stethoscope.

**Blood Pressure.**—Shaw,[13] after criticising Von Basch's sphygmomanometer, describes a tonometer devised by Gärtner, of Vienna, which combines accuracy with simplicity of manipulation. It consists of a pneumatic ring made of metal lined with indiarubber, connected by a lateral tube with a mercurial manometer and an air-ball. The ring is applied to the finger and the latter rendered anæmic; air is pumped into the ring by the ball, and the anæmia thus maintained. By allowing the air to escape gradually the point is reached when the pressure exactly equals the arterial pressure in the digital arteries, and the finger begins to regain its colour.

The pressure required to just produce anæmia is read off from the manometer scale. Shaw holds that such an instrument has a great future, for not only have useful observations been made already in physiological conditions, but in pathological also. So easy is its manipulation that it should furnish the practitioner with an instrument for the daily routine of practice. Van Santvoord[14] holds that the pressure exerted upon an artery by the spring of the sphygmograph is only a measure of the absolute arterial tension when comparison is made between vessels of equal calibre. The writer believes that "the tracing itself will give a far more accurate record of the absolute tension of the vessel than the amount of pressure required to develop it; rapid and extensive variation of tension being indications of great resistance and high absolute tension."

He criticises Von Basch's sphygmomanometer in that it is not easy to determine when pulsation has ceased; the size of the compressed vessel is unknown, the instrument measures only the highest point of pressure existing during the cardiac cycle, and indicates no variation.

**Rhythm and Blood.**—Janeway[15] communicates a case of bradycardia in a young man without any sufficient cause recognised. The pulse-rate on his entrance into the army had been 100 per minute; now it had dropped to 20-60 (usually 30) per minute. It seems to have grown slower gradually. Hoffmann[16] considers tachycardia a symptom of a cerebral disorder (4th vent.), and Vierordt[17] that cyanosis in congenital cardiac affections is due to excessive amount of blood in the cutaneous vessels, and not to an admixture of arterial and venous blood.

Stengal,[18] in studying the condition of the blood in diseases of the cardio-vascular system, has found that in acute endocarditis there is a rapid reduction of red corpuscles (50 to 60 per cent.). Leucocytes are generally increased in number, and polymorphous cells may reach 98·5 per cent. Organisms are frequently found bacteriologically.

In valvular disease the number of red corpuscles may be greatly increased (especially with chronic cardiac disease with continuous slight inadequacy of circulation), and he criticises the theory that this is due to reduced blood pressure through loss of liquid from the blood, especially through the lungs (Grawitz), for he states that these conditions may occur without polycythæmia. He holds that the determination of the corpuscles to the cutaneous vessels plays an important part, and quotes Mitchell, who states that massage is followed by progressive increase in the number of blood corpuscles. This may be due to an increase in viscosity holding the corpuscles back from the circulation.

Pulsus paradoxus, according to Hay,[19] need not be pathological, though it is most marked in cases of indurative anterior mediastinopericarditis. There are other conditions in which it may appear, as in pericarditis, with effusion without mediastinitis, in pleuritic effusion and great inspiratory dyspnœa. In this connection he quotes two cases which tend to show that—(1) It may be relieved by thoracentesis in cases of pleural effusion; that it is associated with low arterial tension—as Sir W. Broadbent points out, a hard-tension pulse hardly reacts to variations, "which produce extraordinary perturbation in the low-tension pulse." (2) That the condition may only be temporary, possibly while the tension is low; on its regaining a more normal condition the pulsus paradoxus ceases. Barr[20] has failed to find anything of importance in the "pulsus paradoxus." He states that it can be produced in health if the individual has a good respiratory pump and low-tension pulse. Hay held that the phenomenon had its main feature in the distinct rhythm, and as such was always pathological. Gullan reminds us that it has been noticed in children when asleep.

**Endocarditis.**—MacCallum and Hastings[21] give a case of acute endocarditis caused by the *Micrococcus Zymogenes*. This was twice isolated in pure culture from the blood during life, and shown on the valvular vegetations and infarcts after death.

Litten[22] classifies endocarditis thus:—

A. Ulcerative E . . (1) Purely Septic.
(2) Secondary to Acute Rheumatism,
Peliosis,
Purpura,
Chorea,
Gonorrhœa,
and differentiates these two groups owing to clinical, anatomical and bacteriological modifications.

B. Benign. This is not idiopathic but secondary to other diseases.

C. Malignant. (*a*) Non-ulcerative, always accompanied by some severe disease, and constitutes a grave danger to life, though prognosis not absolutely bad. Death supervenes when dyspnœa, cyanosis, and cerebral symptoms appear. The gonorrhœal variety is the most favourable to absorption. (*b*) Ulcerative (septic), due to B. Pyogenes Albus, B. Aureus-citreus-streptococcus pyogenes diplococcus, B. Griseus. Always progressive and is a symptom of pyæmia.

Potain[23] records a unique case of gonorrhœal aortitis. Aortitis from gonorrhœa has never been recorded before. The symptoms of this affection are, he says, obscure retro-sternal pains resembling angina pectoris, cough, unsatisfied dyspnœa, palpitation sometimes severe. The temperature may be high, the aortic dulness may be increased, and the subclavian artery raised. The aorta is lengthened and so the curve exaggerated: thus the arch is pushed upwards with its branching vessels, the innominate especially, and so the right subclavian vessel appears above the clavicle.

He reviews the causes of aortitis thus: syphilis, acute febrile disorders (variola, pyæmia, scarlatina, typhoid), acute articular rheumatism, pneumonia, malaria, diphtheria and influenza, and now gonorrhœa must be added to the category.

**Pericarditis.**—Hale White,[24] with relation to the prognosis in pericarditis, states that it is not necessarily bad that a man has his pericardium adherent. The important point is, is there coincident dilatation? If the cardiac apex moves far outwards and the dulness is greatly increased, caution must be exercised in giving a favourable prognosis. Prognosis depends upon (1) the state of the heart and (2) the state of the pericardium. The pericarditis of Bright's disease is the worst form, because the patient has bad tissues. If adhesions take place when the heart is normal in size then no evil results; if when dilated, however, the patient will probably suffer from the effects of backward pressure and venous stasis. Other points of importance are whether much strain is thrown on the heart when weakened by inflammation and whether adhesions prevent its growth, as in children. Merklen[25] gives the notes of an interesting case of "cardiac adhesion," which was watched during its development in a youth of 16. Merklen, remarking on the case, states that the abnormal movements in the precordia, though presumptive, are no direct evidence of an adherent pericardium, for that this occurs in cases where adherency does not exist: it is not to be relied upon—it is only necessary that the heart be enlarged and the lungs in front displaced for this to be produced. It is not seen when there is only dilatation or enfeeblement. Hypertrophy and dilatation are a second presumptive sign. Cardiac adhesion is rarely seen uncomplicated when of rheumatic origin; it is only one of the elements of what might be called a "pancarditis." The chronic pericarditis is answerable in this case for the enormous size of the heart, in the absence of mitral mischief. It is a total enlargement and "is as large as acute pericarditis with effusion." The association of external pericardial adhesions consequent on mediastinal and diaphragmatic pleurisy is not always present, though frequent, with adherent pericardium, but when present it is strong evidence.

[10] Post Graduate, Feb. [11] Treatment, Feb. 8. [12] Med. Chron., May. [13] Med. Rec., Feb. 3. [14] Med. Rec., Feb. 24. [15] Med. Rec., April 28. [16] Lancet, May 26. [17] Idem. [18] Brit. Med. Jour., April 14. [19] Lancet, Feb. 24. [20] Brit. Med. Jour., May 5. [21] Post Graduate, Feb. [22] Brit. Med. Jour., May 5. [23] Med. Bulletin, Feb. [24] Clin. Jour., Mar. 28. [25] La Semaine Medicale, Feb. 21.

